# Optimization of heterologous protein production in Chinese hamster ovary cells under overexpression of spliced form of human X-box binding protein

**DOI:** 10.1186/1472-6750-14-26

**Published:** 2014-04-11

**Authors:** Galina Gulis, Kelly Cristina Rodrigues Simi, Renata Rodrigues de Toledo, Andrea Queiroz Maranhao, Marcelo Macedo Brigido

**Affiliations:** 1 Institute of Biological Sciences, Department of Cell Biology, University of Brasilia, Campus Universitário Darcy Ribeiro, Brasília, DF 70910-900, Brazil

**Keywords:** CHO cells, Heterologous protein production, X-box binding protein, T-REx™ system, Doxycycline

## Abstract

**Background:**

The optimization of protein production is a complex and challenging problem in biotechnology. Different techniques for transcription, translation engineering and the optimization of cell culture conditions have been used to improve protein secretion, but there remain many open problems involving post-translational modifications of the secreted protein and cell line stability.

**Results:**

In this work, we focus on the regulation of secreted protein specific productivity (using a recombinant human immunoglobulin G (IgG)) by controlling the expression of the spliced form of human X-box binding protein (XBP-(s)) in Chinese hamster ovary cells (CHO-K1) under doxycycline (DOX) induction at different temperatures. We observed a four-fold increase in specific IgG productivity by CHO cells under elevated concentrations of DOX at 30°C compared to 37°C, without detectable differences in binding activity *in vitro* or changes in the structural integrity of IgG. In addition, we found a correlation between the overexpression of human XBP-1(s) (and, as a consequence, endoplasmic reticulum (ER) size expansion) and the specific IgG productivity under DOX induction.

**Conclusions:**

Our data suggest the T-REx system overexpressing human XBP-1(s) can be successfully used in CHO-K1 cells for human immunoglobulin production.

## Background

The optimization of the production of secreted proteins, such as therapeutic monoclonal antibodies (mAbs), is still a challenging problem in pharmaceutical biotechnology. Although biopharmaceutical products can be produced by many host cell systems, eukaryotic cells are preferred due to their ability to correctly process and modify human proteins. The primary goal is to establish the ideal combination of a rapid accumulation of productive biomass and the maintenance of cell viability for as long as possible. Many different strategies have been considered for improving both cell viability and the productivity of recombinant proteins, including mAbs. These strategies include physiological optimization and genetic and metabolic engineering [1,2].

The most common problem during the optimization of protein production is an error in protein folding in the endoplasmic reticulum (ER). The inhibition of protein folding activates the unfolded protein response (UPR), which is a signal transduction network. Overcoming UPR is one of the many strategies for optimizing protein productivity. For instance, protein production has been tested under the expression of survival proteins that play important roles in UPR, including B-cell lymphoma protein 2 (bcl-2), B-cell lymphoma-extra-large protein (bcl-XL) [3-5], caspase inhibitors [6] and molecular chaperones/heat shock proteins (HSP70) [7]. The role of the spliced form of X-box binding protein (XBP-1(s)) (which plays an important role in regulation processes, such as physical expansion of the ER, increasing the mitochondrial mass and function, increasing the cell size and enhancing total protein synthesis) in optimizing protein production has also been studied [8]. This approach to increasing the secretion capacity of mammalian cells by overexpressing the transcription factor XBP-1(s) was successful in CHO cells; the production of the secreted proteins alkaline phosphatase (SEAP) and alpha-amylase (SAMY) was enhanced upon XBP-1(s) overexpression [9], as was the production of antibody [10]. However, these studies have shown that using overexpression systems without regulation leads to cell apoptosis due to the accumulation of the produced proteins [11].

To overcome accumulation-induced apoptosis, other strategies have been applied to regulate the protein production, such as the use of induction systems. For instance, tetracycline has been used to optimize the overexpression of glycosyltransferases under the control of the Tet on/off system in CHO cells, but unfortunately, a high expression of glycosyltransferases still led to growth inhibition [12]. Furthermore, interesting work using the same expression system has been conducted to control the overexpression of human transferrin (hTf) in human embryonic kidney (HEK-293) cells. That study found favorable concentrations of tetracycline at which the overexpression of hTf was optimal, but again, the high levels of expression limited the cell viability. Such impairment might have been a consequence of the overexpression of the protein of interest, which might have altered the quality of this cell product or even been toxic to the cells [13]. Some studies have attempted to investigate the effect of the expression of an ER-resident molecular chaperone, protein disulfide isomerase (PDI), on the specific production levels of thrombopoietin (TPO) and antibody (Ab) in Chinese hamster ovary cells. Mohan and colleagues used the Tet-off system (in the absence of tetracycline) to regulate PDI, TPO and Ab expression in CHO cells under doxycycline (DOX; a chemical analogue of tetracycline) induction. However, only a small increase in antibody production was observed, and the production of TPO was not affected by PDI expression [14].

Moreover, the optimization of protein production in CHO cells cultured at different temperatures has been addressed. For instance, lowering the temperature from 37°C to 33°C increased the production of erythropoietin (EPO) by approximately four-fold, but at the same time, a low cultivation temperature suppressed cell growth [15]. In addition, a temperature reduction from 37°C to 33°C in the culture of a CHO cell line producing recombinant human granulocyte/macrophage colony-stimulating factor (CHO-K1-hGM-CSF) led to a reduced growth rate, increased cell viability, improved cellular protein production and decreased cell metabolism [16]. One study on the optimization of protein production at 32°C also demonstrated that the specific growth rate of CHO cells producing human mAb decreased by 30–63% at 32°C compared to 37°C. However, the specific antibody productivity of these cells was significantly enhanced at 32°C [17]. Lowering the cultivation temperature even more, from 37°C to 30°C, caused growth arrest associated with a 1.7-fold increase in the specific production of secreted alkaline phosphatase (SEAP) in CHO cells [18].

**Figure 1:**
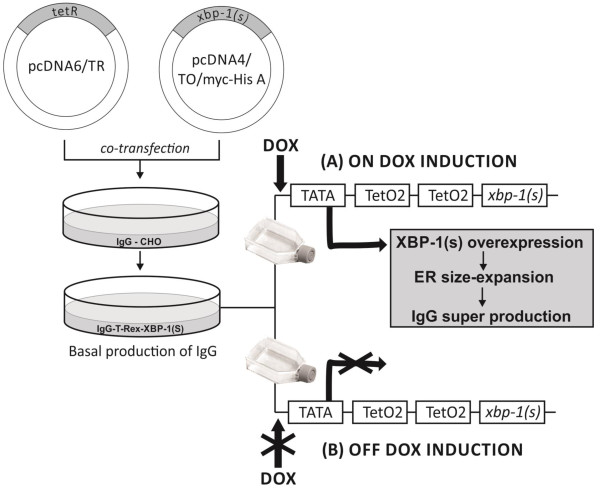
**Schematic representation of the DOX-regulated T-Rex™ overexpression XBP-1(s) system.** The overproduction of IgG as a result of the XBP-1(s) overexpression and ER size expansion under DOX induction (on DOX induction) **(A)**. The repression of XBP-1(s) overexpression and ER size expansion resulted in the repression of overproduction of IgG in the absence of DOX (off DOX induction) **(B)**.

In this context, we attempted to optimize the specific IgG productivity under different culture temperatures and by regulating the overexpression of apoptotic human protein XBP-1(s) using the T-REx™ system (Invitrogen, Carlsbad, CA, USA). The applied T-REx™ system contains a regulatory plasmid (pcDNA6/TR), which encodes the tetracycline repressor, and an inducible expression plasmid (pcDNA™4/TO/myc-His A) with a tetracycline inductor for expression of the gene of the interest (*xbp-1(s)*). Co-transfected together, these plasmids created a network to regulate XBP-1(s) expression under DOX induction. We cloned *xbp-1(s)* into the T-REx™ system to control its expression with DOX. Then, we transfected the obtained T-REx™-XBP-1(s) system into stably IgG-producing CHO cells and selected stable clones of this system expressing IgG-T-REx-XBP-1(s) to control specific IgG productivity under DOX induction (Figure 1). We determined the optimal concentration of DOX and the temperature at which IgG-T-REx-XBP-1(s) cells produced the maximal amount of IgG without a significant inhibition of cell growth. Moreover, cells treated with DOX for seven days recovered viable cell density to the level of non-treated cells after DOX was washed out from the cell system, and their specific IgG productivity dropped to the basal level. Furthermore, we studied the dependence of specific IgG productivity and viable cell density on the overexpression of XBP-1(s) and ER size expansion.

## Methods

### Cell lines and media

The CHO-K1 (ATCC®CCL-61™) and Raji (ATCC®CCL-86™) cell lines were purchased from American Type Culture Collection (ATCC, Manassas, VA, USA). CHO-K1 cells were grown and maintained at 37°C or 30°C with 70% humidity and 5% CO_2_ in HAM F12 media (Gibco, Big Cabin, OK, USA) supplemented with 2% fetal bovine serum (FBS, Gibco, Big Cabin, OK, USA) and were used in experiments on protein production. Raji cells were grown and maintained at 37°C with70% humidity and 5% CO_2_ in RAMP media (Gibco, Big Cabin, OK, USA) supplemented with 10% FBS and were used in FACS direct ligation experiments.

### Plasmids and cloning

pCOMIRES HIL anti-CD20 is a tricistronic vector that encodes both the heavy and the light chains of an anti-CD20 antibody along with a neomycin resistance gene under the control of a synthetic CMV promoter. This vector was transfected into CHO-K1 cells to obtain IgG (anti-CD20)-producing cells. The human *xbp-1(s)* coding sequence was chemically synthesized by GeneScript (Piscataway, NJ, USA). The restriction enzymes *Hind* III and *Bam*H I (Fermentas, Ontario, Canada) were used to obtain the *xbp-1(s)* insert and then clone it into the inducible expression plasmid pcDNA™4/TO/myc-His A from the Invitrogen T-REx™ system (Invitrogen, Carlsbad, CA, USA). This plasmid was used to co-transfect IgG-producing stable clones of CHO cells along with the regulatory plasmid pcDNA6/TR (Invitrogen, Carlsbad, CA, USA). To confirm *xbp-1(s)* cloning, XL1-blue bacterial cells (Stratagene, La Jolla, CA, USA) were transformed with ligated DNA. Ampicillin (Sigma, Ronkonkoma, NY, USA)-selected colonies were isolated and processed for DNA extraction and purification, which was performed using a QIAprep Miniprep Kit (Qiagen, Valencia, CA, USA). Restriction analysis and sequencing (using CMV forward primer 5′-CGCAAATGGGCGGTAGGCGTG-3′ and BGH reverse primer 5′-TAGAAGGCACAGTCGAGG-3′) confirmed the cloning of the *xbp-1(s)* insert.

### Transfection with pCOMIRES anti-CD20 DNA (IgG-encoding plasmid) into CHO cells and generation of stable IgG-producing cells

The transfection of pCOMIRES HIL anti-CD20 plasmid (encoding an anti-CD 20 (IgG) antibody, a secretable protein with molecular weight 150 kDa (two light chains, each with molecular weight 25 kDa, and two heavy chains, each with molecular weight 50 kDa)) into CHO cells was performed using a PolyPlus (JetPrime, New York, NY, USA) kit in six-well test plates (TPP, San Diego, CA, USA) according to the manufacturer’s instructions. The clones harboring the pCOMIRES HIL anti-CD20 transgene were selected from a mixed population by the single-cell dilution method. Geneticin (Roche, Gaillard, France) was used for selection at 800 μg/mL.

### Transfection with the T-REx™ -XBP-1(s) system into stable IgG-producing clones of CHO cells and generation of stable double clones (IgG-T-REx-XBP-1(s) cells)

The co-transfection of T-REx-*xbp-1(s)* plasmid (encoding a spliced form of human apoptotic XBP-1 protein with predicted molecular weight 40 kDa) along with regulatory plasmid pcDNA6/TR into one of the stable IgG-producing clones was performed using a PolyPlus (JetPrime, New York, NY, USA) kit according to the manufacturer’s instructions in six-well test plates (TPP, San Diego, CA, USA). Blasticidin (Sigma, Ronkonkoma, NY, USA) and Zeocin (Sigma, Ronkonkoma, NY, USA) were added to a final concentration of 0.5 μg/mL and 50 μg/mL, respectively. The selective markers encoded by regulatory plasmid pcDNA6/TR and expression plasmid pcDNA™4/TO/myc-His A are against blasticidin and Zeocin, respectively.

### Doxycycline induction

Selected IgG-T-REx-XBP-1(s) cells (after the first transfection, IgG clones; after the second, co-transfection, T-REx-XBP-1(s) clones) were induced by DOX at different concentrations: 0 μg/mL for control, 0.1 μg/mL, 0.5 μg/mL and 1 μg/mL. We used these concentrations because we found out that 5 μg/ml and 7.5 μg/ml of doxycycline completely inhibits cells growth for clones and wild type CHO-K1 cells. DOX induction was performed 24 hr after IgG-T-REx-XBP-1(s) cells seeding at a uniform cell density (0.5 × 10^5^ cells/mL) in tissue culture flasks (75 cm^2^, TPP, San Diego, CA, USA) and then incubated for seven days at 37°C or 30°C. All cultures reached at least 80% under these conditions. Samples were collected for viable cell density, Ab detection by ELISA, nuclear extract isolation and ER staining. Half of the cells in each group continued to grow for seven more days in DOX-free medium after DOX wash-out. Independently, IgG-T-REx-XBP-1(s) cells were incubated for 42 days at 30°C (150 cm^2^ flasks, TPP, San Diego, CA, USA) with or without 1 μg/mL DOX. In all DOX induction experiments, DOX was added (at an appropriate concentration) every three days to the cell culture. Induction experiments were performed twice in duplicate (four independent culture samples per group).

### Viability assay

The viable cell density of the IgG-T-REx-XBP-1(s) cells were tested under different DOX concentrations (0 μg/mL, 0.1 μg/mL, 0.5 μg/mL or 1 μg/mL) every day during seven days of cell growth at 37°C and 30°C. Seeding was performed at a uniform cell density (0.06 × 10^5^ cells/mL) in six-well tissue culture plates (TPP, San Diego, CA, USA). At the seventh day all cultures reached at least 80% under these conditions. In addition, the viable cell density of IgG-T-REx-XBP-1(s) cells was tested on the seventh day of growth with DOX and on the seventh day after wash-out in DOX-free medium. In addition, IgG-T-REx-XBP-1(s) cells were tested every seventh day during 42 days of cell growth under 1 μg/mL DOX (or 0 μg/mL as control) at 30°C. The viable cell density was measured using the trypan blue (Sigma, Ronkonkoma, NY, USA) exclusion method with a hemocytometer and light microscope for manual cell counting. Every viable cell density experiment was performed twice in duplicate (single determination from each of two independent culture samples per group in two independent experiments).

### ELISA

The supernatants of IgG-T-REx-XBP-1(s) cells in the presence or absence of DOX were collected every seventh day of 37°C or 30°C growth for two weeks or every seventh day for six weeks and processed for analysis by enzyme-linked immunosorbent assay (ELISA) (duplicate determination from each of two independent culture samples per group in two independent experiments). The Lunc/Maxisorp Immunoplate (Thermo Scientific, Waltham, MA, USA) was incubated with primary antibody (goat anti-human IgG (H + L), 1:3000 dilution; Thermo Scientific, Waltham, MA, USA) and blocked with 3% fat-free dehydrated milk solution. After blocking and washing the plate, the supernatants were applied to the plate and incubated for 2 hr. The plate was washed again, and secondary antibody (anti-human IgG Fc-specific, alkaline phosphatase-conjugated, produced in goat, 1:3000 dilution; Sigma, Ronkonkoma, NY, USA) was applied for 1 hr. The plate was washed again, and at the end of the procedure, the signal of absorbance was read at 405 nm by a microplate reader (ELx800 96-well Microplate Reader, MTX Lab Systems, Inc., Vienna, VA, USA) after 4-Nitrophenyl phosphate disodium salt solution (pNPP) (Invitrogen, Carlsbad, CA, USA) addition. In addition, human IgG (whole molecule; Thermo Scientific, Waltham, MA, USA) was used in different concentrations as a control on the same plate.

### Isolation and purification of produced proteins

The IgG produced under different temperature conditions by IgG-T-REx-XBP-1(s) cells was purified on the HiTrap™ Protein A HP 1 mL (GE Life Sciences, Pittsburgh, PA, USA) column. The column was first equilibrated with 10 mL Protein A IgG Binding Buffer (Thermo Scientific, Waltham, MA, USA) at a rate of 1 mL/min. Then, the supernatant from IgG-T-REx-XBP-1(s) cells was applied to the equilibrated column. The column was washed with 30 mL Protein A IgG Binding Buffer (Thermo Scientific, Waltham, MA, USA). Then, the protein was eluted with 50 mL IgG Elution Buffer (Thermo Scientific, Waltham, MA, USA), and 2 mL per fraction was collected. Fractions were neutralized with Tris–HCl pH 9.0. The Ab present in the fractions was immunodetected in a dot blot assay. Five microliters of each fraction was directly pipetted onto a nitrocellulose Hybond-C Extra membrane (Amersham® Bioscience, Piscataway, NJ, USA). The membrane was blocked with 3% fat-free milk solution and incubated with anti-human IgG (Fc-specific, alkaline phosphatase-conjugated, produced in goat, 1:3000 dilution) (Sigma, Ronkonkoma, NY, USA), and the proteins were revealed using a BCIP/NBT substrate Kit (Invitrogen, Carlsbad, CA, USA). The Ab-containing fractions were selected for dialysis, which was performed using a Centricon YM-50 (Amicon Bioseparations, Billerica, MA, USA) in PBS buffer (10 mM NaH_2_PO_4_, 137 mM NaCl, 2.7 mM KCl, pH 7.4).

### Western blotting

Anti-CD20 antibody was also detected by western blotting. Five hundred nanograms of IgG sample was loaded in each well of a Bis-Tris gel (NuPAGE® Novex 4-12% Bis-Tris Gel, Invitrogen, Carlsbad, CA, USA) and separated by sodium dodecyl sulfate–polyacrylamide gel electrophoresis (SDS-PAGE) according to the manufacturer’s instructions. The proteins were transferred to the Hybond-C Extra nitrocellulose membrane (Amersham® Bioscience, Piscataway, NJ, USA) and blocked in 3% fat-free milk PBS solution. The immunodetection was performed using anti-human IgG (Fc-specific, alkaline phosphatase-conjugated, produced in goat (1:3000 dilution) (Sigma, Ronkonkoma, NY, USA) with a BCIP/NBT substrate Kit™ (Invitrogen, Carlsbad, CA, USA).

XBP-1(s) was also probed by western blotting. The nuclear extracts from the IgG-T-REx-XBP-1(s) cells were prepared as described by Becker and colleagues [10]. Briefly, the nuclear extracts were prepared from 5×10^6^ cells/per sample and equal volumes of nuclear extracts were loaded into a Bis-Tris gel (NuPAGE® Novex 4-12% Bis-Tris Gel, Invitrogen, Carlsbad, CA, USA), and SDS-PAGE was performed according to the manufacturer’s instructions. Samples were transferred to the Hybond-C Extra nitrocellulose membrane (Amersham® Bioscience, Piscataway, NJ, USA), and after blocking with 3% fat-free milk PBS solution, rabbit anti-human-XBP-1(s) (1:1000 dilution; Sigma, Ronkonkoma, NY, USA) was added, followed by alkaline phosphatase-conjugated anti-rabbit IgG incubation (1:1000 dilution; Sigma, Ronkonkoma, NY, USA). The proteins were revealed using a BCIP/NBT substrate Kit™ (Invitrogen, Carlsbad, CA, USA).

### Fluorescence-activated cell sorting (FACS)-ER staining

The IgG-T-REx-XBP-1(s) cells that were grown for seven days under DOX induction and those that were grown for one more week after wash-out were collected at 3×10^5^ cells/per staining and washed with HBSS buffer (140 mM NaCl, 4.7 mM KCl, 1 mM MgCl_2_, 1.5 mM CaCl_2_, 10 mM glucose, 10 mM HEPES, pH 7.4). After washing with HBSS buffer, the cells were labeled with 250 nM of ER-Tracker™ Green Dye (ER-Tracker™ Green Dye for Live-Cell Endoplasmic Reticulum, Molecular Probes, Invitrogen, Carlsbad, CA, USA) according to the manufacturer’s manual. The samples were washed again with HBSS buffer and analyzed using a BD FACS Verse flow cytometer (BD Bioscience, San Jose, CA, USA). Ten thousand events were collected per sample using no gate for acquisition. The dead cells were not excluded in the analysis. We used BD FACSuite to data acquisition. The experiment was performed twice in duplicate.

### FACS direct ligation assay

Raji cells were grown for five passages as described above, collected and resuspended in 1 part RAMP media with 10% FBS and 1 part FACS buffer (PBS supplemented with 2% FBS) at 3×10^6^ cells/well in a 96-well plate (TPP, San Diego, CA, USA). After centrifugation, the cells were blocked with FcR blocking reagent (MACS, Biotec, Bergisch Gladbach, Germany) on ice for 30 minutes according to the manufacturer’s instructions. Purified and dialyzed IgG samples, which were produced by IgG-T-REx-XBP-1(s) cells at 37°C and 30°C, and commercial IgG (rituximab, MabThera, Genetech Inc., South San Francisco, CA, USA) as a positive control were added at 100 ng per well. Samples were incubated on ice for 1 hr and centrifuged after the addition of FACS buffer. The cells were washed twice with FACS buffer and incubated with mouse FITC anti-human IgG (BD Pharmingen™, BD Biosciences, San Jose, CA, USA) according to the manufacturer’s manual. The cells were incubated on ice for 30 minutes in the dark, washed again twice with FACS buffer and processed for fluorescence intensity measurements using a BD FACS Verse flow cytometer (BD Bioscience, San Jose, CA, USA). Each experiment was performed twice in duplicate.

## Results and discussion

### Viability and IgG production under induction with DOX in IgG-T-REx-XBP-1(s) cells cultivated at 37°C and 30°C

**Figure 2:**
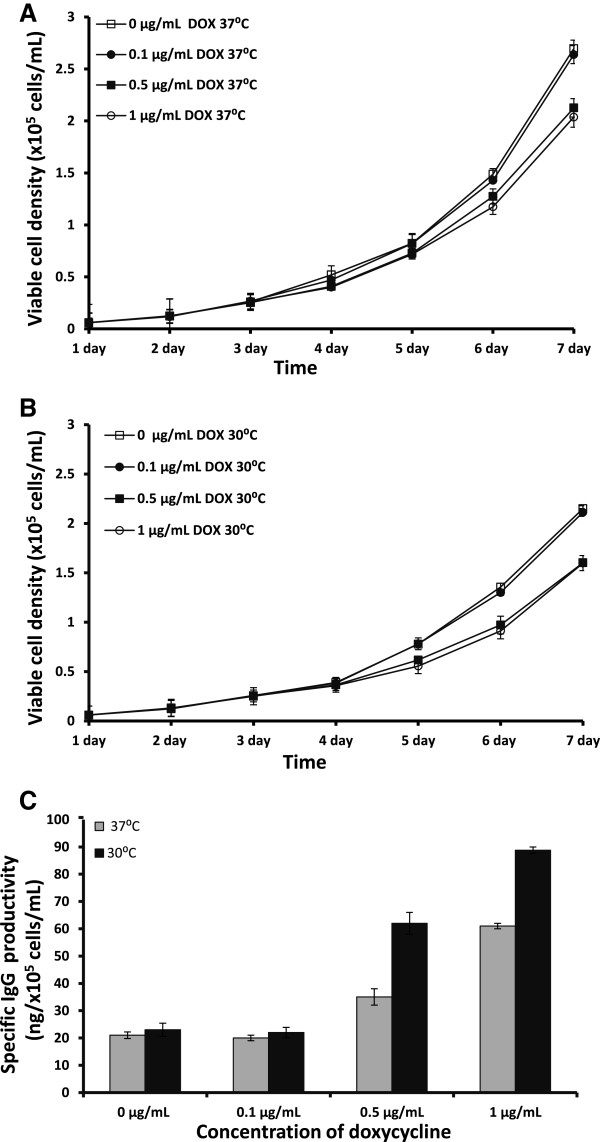
**Viable cell density of IgG-T-Rex-XBP-1(s) cells cultured at 37°C (A) or 30°C (B) and their specific IgG productivity at 37°C and 30°C (C) under induction with 0 μg/mL (control), 0.1 μg/mL, 0.5 μg/mL and 1 μg/mL DOX.** Error bars represent the standard deviation of the mean of two readings from each of two independent culture samples per group in two independent experiments, n = 4.

To establish a DOX-regulated XBP-1(s) cell line, we first created stably IgG-producing clones of CHO cells by transfecting the pCOMIRES HIL anti-CD20 plasmid into CHO-K1 cells. Then, IgG-CHO clones selected with 800 μg/mL of Geneticin were co-transfected with the T-REx-XBP-1(s) system and processed for second selection using 0.5 μg/mL of blasticidin and 50 μg/mL of Zeocin. From these double clones harboring both pCOMIRES HIL anti-CD20 and T-REx-XBP-1(s) system plasmids, we chose one out of 20 for DOX induction at different concentrations (0 μg/mL (control), 0.1 μg/mL, 0.5 μg/mL or 1 μg/mL) and grew them at 37°C or 30°C. Every day, cells were collected to monitor viable cell density. We found that the viable cell density of the IgG-T-REx-XBP-1(s) cells grown at 37°C under 1 μg/mL or 0.5 μg/mL DOX was slightly lower compared to the control and to the cells with 0.1 μg/mL DOX induction (Figure 2A). Moreover, the viable cell density of IgG-T-REx-XBP-1(s) cells grown at 37°C without DOX or with 0.1 μg/mL DOX was 1.25-fold higher compared to the cells grown at 30°C (Figure 2A and 2B). These data agree with several previous studies [15-18]. Moreover, the viable cell density of IgG-T-REx-XBP-1(s) cells grown at 37°C under 1 μg/mL DOX induction was increased by 20.5% compared to cells incubated at 30°C under the same DOX concentration (Figure 2A and 2B). Thus, IgG-T-REx-XBP-1(s) cells grew more slowly at 30°C with or without DOX compared to those at 37°C.

The supernatants from all cells were collected after seven days of induction and tested by ELISA to determine their IgG yields. The specific IgG productivity depended on the concentration of DOX: under 0.5 μg/mL and 1 μg/mL DOX, the increase of specific IgG productivity was 40% and 66%, respectively, compared to the basal level of specific IgG productivity (0 or 0.1 μg/mL DOX) at 37°C (Figure 2C). These data demonstrate that IgG-T-REx-XBP-1(s) cells produced three-fold more IgG compared to untreated cells, even at low viable cell density. At 0.1 μg/mL DOX, there was no improvement in specific IgG productivity at 37°C. Moreover, the data from ELISA indicate that protein production in the cells incubated at 30°C increased four-fold and three-fold under 1 μg/mL and 0.5 μg/mL DOX, respectively. Once more, induction at a low concentration of DOX (0.1 μg/mL) did not increase specific IgG productivity at 30°C, as above at 37°C. In contrast, the specific IgG productivity by IgG-T-REx-XBP-1(s) cells at 30°C increased by 31.5% and 43.5% compared to induction at 37°C under 1 μg/mL and 0.5 μg/mL DOX concentrations, respectively (Figure 2C). However, we did not detect any effect of low temperature on specific IgG productivity *per se* (without the induction of DOX). Tigges and Fussenegger [9] reported the same lack of effect in CHO cells expressing SEAP, whereas other authors reported an increase in the production of different proteins at low temperature and with no inductor [15-18]. These deviations in experimental results may be due to differences in the proteins and cell lines used in these studies. In conclusion, our data demonstrate a successful improvement of specific IgG productivity using 1 μg/mL DOX in IgG-T-REx-XBP-1(s) cells at 30°C.

### Effect of XBP-1(s) expression and ER size expansion on protein production in IgG-T-REx-XBP-1(s) cells

**Figure 3:**
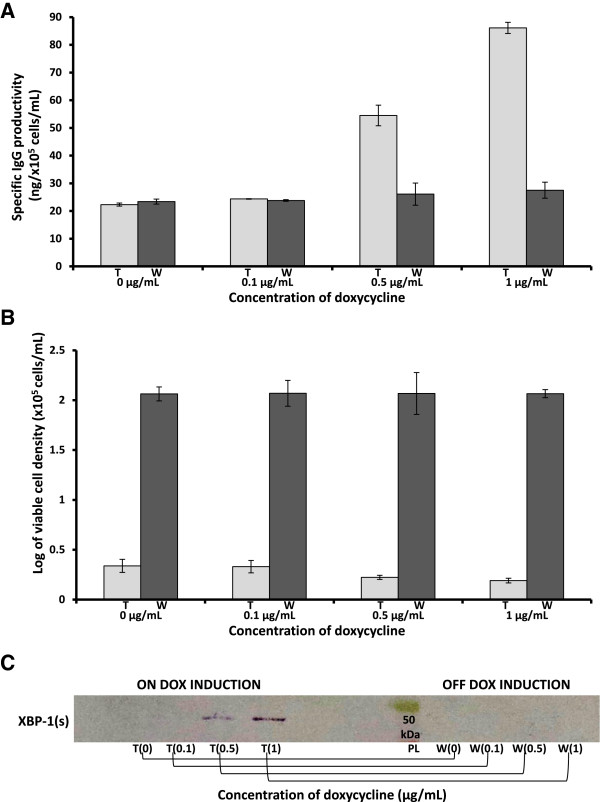
**The specific IgG productivity (A), log of viable cell density (B) and western blot analysis of nuclear extracts (C) from the IgG-T-Rex-XBP-1(s) cells grown at 30°C under 0 μg/mL, 0.1 μg/mL, 0.5 μg/mL or 1 μg/mL DOX for seven days (treated, t) and from the same cells seven days after DOX wash-out (washed, w).** PL, protein ladder. Error bars represent the standard deviation of the mean of double determination from each of two independent culture samples per group in two independent experiments, n = 4.

To test the hypothesis that XBP-1(s) expression and ER size expansion indirectly regulate protein production, IgG-T-REx-XBP-1(s) cells were incubated under different concentrations of DOX for seven days at 30°C, and then the same cells were washed with DOX-free medium and incubated for seven more days at the same temperature in fresh DOX-free medium. The supernatant was collected before wash-out and after seven days of incubation in DOX-free medium and processed for ELISA. ELISA showed that specific IgG productivity by cells with DOX (first seven days) increased in a DOX concentration-dependent manner. The specific IgG productivity by cells incubated with 1 μg/mL DOX and 0.5 μg/mL DOX reached four-fold and three-fold that of the untreated cells (treated (t), Figure 3A). In addition, ELISA demonstrated that after DOX removal, the specific IgG productivity returned to the basal level (washed, (w) Figure 3A). Moreover, viability analysis indicated that the growth of the IgG-T-REx-XBP-1(s) cells under different concentrations of DOX was slightly inhibited (first seven days, treated (t), Figure 3B) and then restored to the same level (washed (w), Figure 3B) as cells that had never been exposed to DOX (0 μg/mL (t or w), Figure 3B). In addition, IgG-T-REx-XBP-1(s) cells were used to prepare nuclear extracts, which were analyzed by western blotting for the immune detection of XBP (s). Human XBP-1 (s) was overexpressed in a DOX concentration-dependent manner (first seven days, on DOX induction, treated (t) Figure 3C), but it was absent in cells that were washed and incubated in DOX-free medium for seven days (last seven days, off DOX induction, washed (w), Figure 3C).

**Figure 4:**
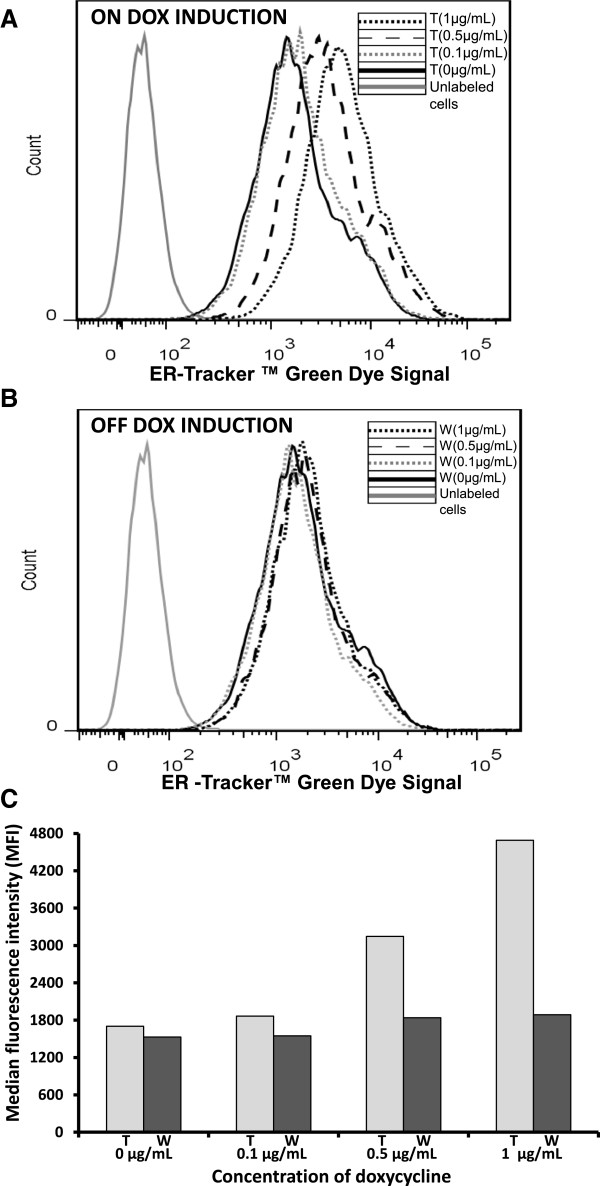
**FACS analysis.** The samples were run through the flow cytometer until 10.000 events were collected using no gate. The IgG-T-Rex-XBP-1(s) cells were grown under induction with 0 μg/mL, 0.1 μg/mL, 0.5 μg/mL or 1 μg/mL DOX (on DOX induction, treated (t)) for seven days at 30°C. Then, cells from each group were washed with DOX-free media and grown in DOX-free media for seven more days at 30°C (off DOX induction, washed (w)). The cells from each group were stained with ER-tracker™ green dye, and cell counts vs. ER-Tracker™ signal from IgG-T-REx-XBP-1(s) cells were measured (on DOX induction, treated (t) **(A)** and off DOX induction, washed (w) **(B)**). Median fluorescence intensity (MFI) of ER-Tracker™ Green Dye from IgG-T-REx-XBP-1(s) cells (on DOX induction, treated (t) and off DOX induction, washed (w) **(C)**).

ER expansion was also observed by flow cytometry. The fluorescence change of ER-Tracker™ was used as a measure of ER size expansion. In this analysis, cell staining was more intense in samples treated with 1 μg/mL or 0.5 μg/mL DOX for seven days (on DOX induction, treated (t), Figure 4A) than in those treated with 0.1 μg/mL (on DOX induction, treated (t), Figure 4A) or 0 μg/mL or those that were washed out (w), Figure 4B). Moreover, the signal from the washed out cells was equal among different conditions (off DOX induction, washed (w), Figure 4B), which indicated that DOX was responsible for a cascade of processes leading to ER size expansion. In addition, measurements of the median fluorescence intensity (MFI, Figure 4C) obtained from FACS analysis showed that the MFI of the cells treated with 1 μg/mL or 0.5 μg/mL DOX was 2.7-fold or 1.85-fold higher, respectively, than the MFI of non-treated cells or washed out cells (Figure 4C). Thus, cells under DOX induction and low temperature grew more slowly but, at the same time, exhibited a greater increase in specific IgG productivity. Our data also demonstrate that the wash-out of DOX from the cells restored their viable cell density but reduced their specific IgG productivity to basal levels. Taken together, our results indicate the optimal conditions for specific IgG productivity under DOX induction. XBP-1(s) was overexpressed under induction with DOX, which led to the ER size expansion, and this resulted in an increase of specific IgG productivity/secretion. Our findings corroborate the data obtained by Tigges and Fussenegger, who reported an increased production of SEAP and SAMY under the expression of XBP-1(s) and the expansion of ER and Golgi [9].

### Binding activity of the recombinant proteins produced at different temperatures

**Figure 5:**
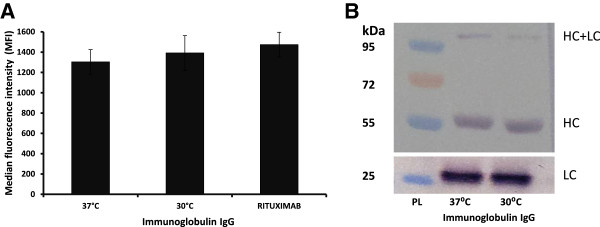
**FACS and Western analyses.** Median fluorescence intensity measurements of mouse FITC anti-human IgG ligated to the tested IgG (produced by IgG-T-REx-XBP-1(s) cells under DOX induction at 37°C and 30°C (and rituximab, control) that were previously incubated with Raji cells) **(A)**. Western blot analysis of IgG produced by IgG-T-REx-XBP-1(s) cells at 37°C and 30°C under DOX induction (PL, protein ladder) **(B)**.

To assess its binding activity, the recombinant IgG produced by cells at different temperatures (37°C and 30°C) was purified and tested for its ability to recognize CD20 at the cell surface of Raji cells that were subjected to flow cytometry analysis. FACS analysis indicated no significant difference in the binding activity of IgG produced by IgG-T-REx-XBP-1(s) cells at different temperatures under DOX induction from commercial IgG (rituximab) (Figure 5A). Moreover, protein samples obtained from the IgG-T-REx-XBP-1(s) cells at different temperatures under DOX induction were submitted to western blotting analysis, and the results did not suggest any differences in structural integrity of IgG produced at different temperatures (Figure 5B). These data support the use of low-temperature culture conditions under induction by DOX to increase protein production without eliminating the binding activity and structural integrity of the protein of interest.

### Establishing a stable protein-producing cell line

**Figure 6:**
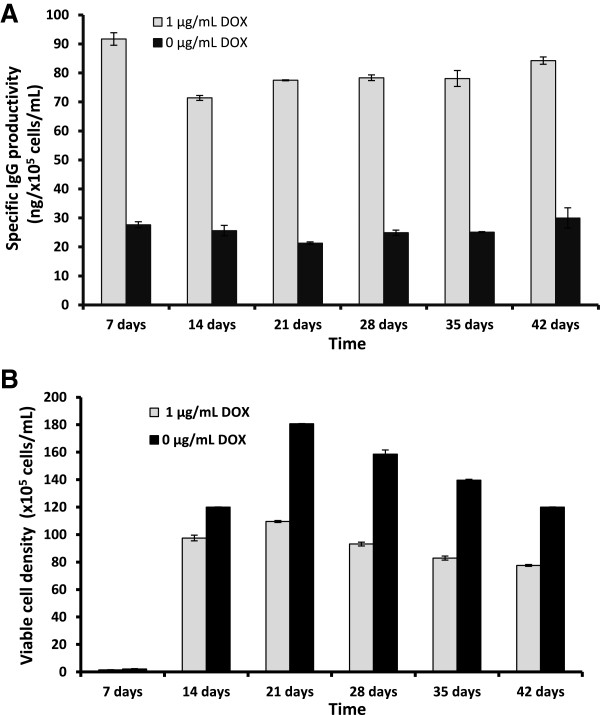
**Specific IgG productivity (A) and viable cell density (B) of the IgG-T-REx-XBP-1(s) cells under 1 μg/mL DOX induction (0 μg/mL DOX, control) for 42 days at 30°C.** Error bars represent the standard deviation of the mean of double determination from each of two independent culture samples per group in two independent experiments, n = 4.

To establish a stable cell line, IgG-T-REx-XBP-1(s) cells were seeded and grown at 30°C under 1 μg/mL DOX (or 0 μg/mL DOX as control) for 42 days. The supernatant was collected every seventh day and submitted to ELISA, which showed stable specific IgG productivity around an average of 80.2 ng/10^5^ cells/mL by cells incubated with DOX compared to the basal level (25.7 ng/10^5^ cells/mL in cells without DOX) (Figure 6A). Moreover, the viable cell density of IgG-T-REx-XBP-1(s) cells under 1 μg/mL DOX was lower than cells without DOX for 42 days (Figure 6B), which allowed cells to be kept in the same culture flask without passaging to new flasks. The described approach might be useful for the production of recombinant secreted proteins, as cells growing under the combination of DOX induction and low temperature multiply more slowly but are more productive.

## Conclusion

Many studies have been published on improving recombinant protein production. In general, the published data suggest that the optimization of the production of specific target proteins requires specifically adjusted growth conditions and a carefully chosen cell line. In the present study, we optimized the conditions for IgG (human anti-CD20) specific productivity in CHO-K1 cells. We showed that the combination of low temperature (30°C) and XBP-1(s) overexpression regulated by DOX induction significantly improved anti-CD20 specific productivity: under 1 μg/mL DOX treatment, specific IgG productivity was increased by 32% compared to the cells grown under the same concentration at 37°C and 74% compared to the cells grown without DOX induction at 37°C or 30°C. Moreover, the results of our study indicate the direct dependence of specific IgG productivity on the concentration of DOX (under 0.5 μg/mL, the increase was 2.7-fold, and under 1 μg/mL, the increase was 3.9-fold), which allows for the precise regulation of specific IgG productivity in CHO-K1 cells. In addition to the concentration dependence, we demonstrated the possibility of returning the specific IgG over productivity to the basal level of specific productivity by removing DOX. This step also restored the viable cell density, which permitted the cells to overcome the problem of accumulation of the target protein. In the production of proteins, it may be possible to use the T-Rex-XBP-1(s) system to turn up and down the production of protein, repeating this cycle several times to accumulate higher amounts of target protein without a loss of cell viability. We also observed a DOX concentration-dependent relationship involving XBP-1(s) overexpression (western analysis), ER size expansion (FACS measurements) and specific IgG productivity (ELISA). Finally, our data demonstrate that it is possible, under DOX induction at low temperature, to produce a target protein for an extended period of time. Taken together, our data suggest the T-REx-XBP-1(s) system can be used in CHO-K1 cells for human immunoglobulin production.

## Abbreviations

Ab: Antibody; bcl-2: B-cell lymphoma protein 2; bcl-XL: B-cell lymphoma-extra-large protein; CHO-K1: Chinese hamster ovary cells; CHO-K1-hGM-CSF: CHO cell line producing recombinant human granulocyte/macrophage colony-stimulating factor; DOX: Doxycycline; ELISA: Enzyme-linked immunosorbent assay; EPO: Erythropoietin; ER: Endoplasmic reticulum; FACS: Fluorescence-activated cell sorting; HEK-293: Human embryonic kidney cells 293; HSP70: Heat shock proteins 70; hTf: Human transferrin; IgG: Human immunoglobulin G; mAbs: Monoclonal antibodies; MFI: Median fluorescence intensity; PDI: Protein disulfide isomerase; pNPP: 4-Nitrophenyl phosphate disodium salt solution; SAMY: alpha-amylase; SEAP: Secreted alkaline phosphatase proteins; SDS-PAGE: Sodium dodecyl sulfate–polyacrylamide gel electrophoresis; Tet: Tetracycline; TPO: Thrombopoietin; UPR: Unfolded protein response; XBP-(s): Spliced form of human X-box binding protein.

## Competing interests

The authors declare that they have no competing interests.

## Authors’ contributions

GG, AQM and MMB designed the study, interpreted the results and wrote the manuscript. GG, KCRS and RRT performed the experiments and interpreted the results. All authors read and approved the final manuscript.
